# Lymphatic Vascularisation and Involvement of Lyve-1^+^ Macrophages in the Human Onchocerca Nodule

**DOI:** 10.1371/journal.pone.0008234

**Published:** 2009-12-09

**Authors:** Tarik Attout, Achim Hoerauf, Gaëlle Dénécé, Alexander Yaw Debrah, Yeboah Marfo-Debrekyei, Michel Boussinesq, Samuel Wanji, Valérie Martinez, Sabine Mand, Ohene Adjei, Odile Bain, Sabine Specht, Coralie Martin

**Affiliations:** 1 USM 307, Parasitologie comparée et Modèles expérimentaux, Muséum National d'Histoire Naturelle, Paris, France; 2 Institute for Medical Microbiology, Immunology and Parasitology, University Clinic Bonn, Bonn, Germany; 3 Kumasi Centre for Collaborative Research in Tropical Medicine (KCCR), Kumasi, Ghana; 4 IRD, UMR-1045, Montpellier, France; 5 Research Foundation in Tropical Diseases and Environment, Buea, Cameroon; 6 Department of Biochemistry and Microbiology, University of Buea, Buea, Cameroon; 7 Service de Médecine Interne et Immunologie Clinique, Hôpital Antoine Béclère, Clamart, France; New York University, United States of America

## Abstract

Onchocerciasis, caused by the filarial nematode *Onchocerca volvulus*, is a parasitic disease leading to debilitating skin disease and blindness, with major economic and social consequences. The pathology of onchocerciasis is principally considered to be a consequence of long-standing host inflammatory responses. In onchocerciasis a subcutaneous nodule is formed around the female worms, the core of which is a dense infiltrate of inflammatory cells in which microfilariae are released. It has been established that the formation of nodules is associated with angiogenesis. In this study, we show using specific markers of endothelium (CD31) and lymphatic endothelial cells (Lyve-1, Podoplanin) that not only angiogenesis but also lymphangiogenesis occurs within the nodule. 7% of the microfilariae could be found within the lymphatics, but none within blood vessels in these nodules, suggesting a possible route of migration for the larvae. The neovascularisation was associated with a particular pattern of angio/lymphangiogenic factors in nodules of onchocerciasis patients, characterized by the expression of CXCL12, CXCR4, VEGF-C, Angiopoietin-1 and Angiopoietin-2. Interestingly, a proportion of macrophages were found to be positive for Lyve-1 and some were integrated into the endothelium of the lymphatic vessels, revealing their plasticity in the nodular micro-environment. These results indicate that lymphatic as well as blood vascularization is induced around *O. volvulus* worms, either by the parasite itself, *e.g.* by the release of angiogenic and lymphangiogenic factors, or by consecutive host immune responses.

## Introduction

Onchocerciasis is caused by the filarial nematode *Onchocerca volvulus*. This disease is an important cause of visual impairment in African countries. Once the infective larvae penetrate into the skin during a blood meal of black fly vectors (*Simulium* spp.) they develop into male and female adult worms, residing in subcutaneous nodules (onchocercomas) and producing large numbers of microfilariae. In these nodules adult females can reach 70 cm in length. Although males can be observed alongside females within nodules, they are never identified alone in onchocercomas. This is also observed in cattle onchocercomas [1], [2] suggesting that nodules are induced by female filariae [3]. A particularity of onchocerciasis is the localisation of microfilariae in the dermis and not in the blood, unlike many other filarial species. Besides the skin, microfilariae are also able to migrate into the eye, where they can induce serious lesions.

The majority of infected individuals has a hyporeactive form of the disease (generalized onchocerciasis), characterised by a mixed T helper type (Th) 1, Th2 and T regulator (Tr) 1 response, induced by the filarial as well as *Wolbachia* bacteria [4]. These endosymbionts are able to induce a pro-inflammatory Th1 response recruiting neutrophils and activating macrophages, counterbalancing the filarial induced Th2 response [5], [6], [7], [8].

Macrophages are the predominant cell type in the nodule and have a regulatory role in antigenic stimulation [9], [10]. They also act on microfilariae fitness as those with morphological alterations or microfilariae after ivermectin treatment were nearly always surrounded by adherent macrophages [11], [12], [13]. Epithelioid cells and giant cells can be found near the worms. Other cells in the nodule include lymphocytes, polymorphonuclear neutrophils, eosinophils, plasma cells, and mast cells [12], [14], [15], [16]. The core of the nodule is limited by a fibrous outer layer which is attached to the skin, deep fasciae, bones or joints [17]. As in solid tumours, there is evidence that the extent of nodule formation is associated with new blood vascularisation [18], [19] which enables infiltration of host immune cells but could also supply nutrients to the worms.

Microfilariae are released by female worms in the inflammatory core of the nodule from where they can migrate long distances through the body of *Onchocerca* patients [20], [21]. In cervids infected with *Onchocerca* species microfilariae accumulated in the ears whereas the adult worms were present in the legs [22]. A role for the lymphatic system in microfilariae migration processes has been suggested [23], [24] but, to date, lymphangiogenesis in the nodule and dissemination of microfilariae from these vessels have not been investigated, due to technical limitations, such as the more recent development of specific markers.

Using specific antibodies we could confirm the presence of blood and lymphatic vessels in onchocercomas. Both of them were largely distributed within the nodules and present in close proximity to the worms. Almost 7% of microfilariae were found within lymphatics of the nodule, however they were not present in the lymphatics of the skin. In addition, we identified the presence of angio/lymphangiogenic factors needed for vessel formation in onchocercomas. These were characterized by the gene expression of chemokine (C-X-C motif) ligand 12 (CXCL12), chemokine (C-X-C motif) receptor 4 (CXCR4), vascular endothelial growth factor C (VEGF-C), Angiopoietin-1 and Angiopoietin-2 in the nodules of patients. A striking finding in this study was the presence of macrophages expressing the lymphatic endothelial cell marker Lyve-1 within the lymphatic vessels of the nodules suggesting a contribution of macrophages to the neovascularisation process and thus a new role of these cells in onchocercoma development.

Knowledge of the induction and growth of nodules, especially of the involvement of the vascular system in these processes and its contribution to parasite fitness and survival, might be helpful for identification of new drug targets against onchocerciasis. Even if efforts are made to test new drugs against human *O. volvulus* and bovine *Onchocerca* spp. (moxidectin and antibiotics targeting the *Wolbachia* endosymbionts of the parasites), onchocerciasis control relies presently on a single drug, ivermectin. In this context, targeting angiogenesis and lymphangiogenesis in onchocercomas may provide new mechanisms either to supply chemotherapy in closer contact to worms, or to prevent the expansion of the nodule by blocking the entry of inflammatory cells through the destruction of the blood system.

## Results

### Abundance of Lymphatic Vessels in Onchocercoma

**Figure 1:**
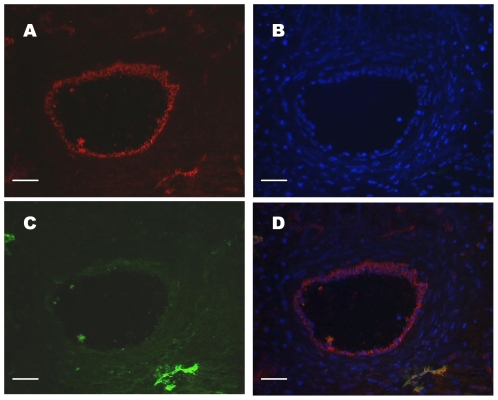
Blood and lymphatic identification in onchocercoma. Five-micrometer serial sections were stained with (A) CD31 or (C) Lyve-1; (B) DAPI nuclear counterstaining. (D) Merging of A, B and C. Number of analysed nodules = 5. Scale: A, B, C, D: bar = 25 µm.

**Figure 2:**
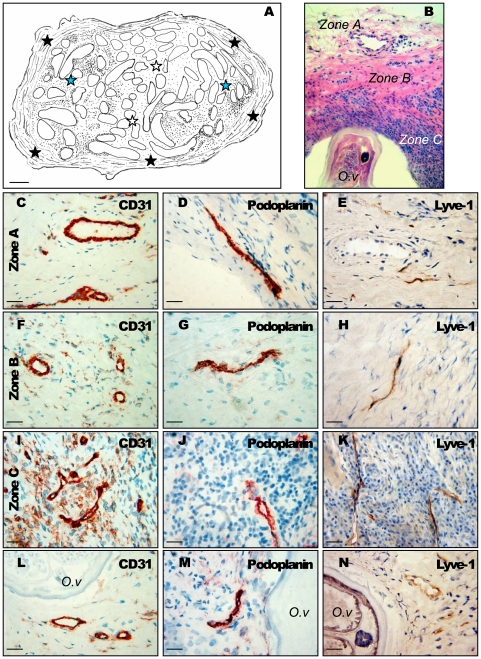
Vascular organisation of onchocercoma. (A) The different zones in nodules: zone A = the outer fibrovascular capsule (black stars), zone B = the inner adult worm bundle with surrounding extracellular matrix interspersed with solitary cells (white stars), zone C = a dense cellular infiltrate surrounding and in contact with the worm (blue stars). (B–M) The blood and lymphatic system in the nodules: sections of onchocerca nodules were stained for CD31 (B, E, H, K) to detect blood and lymphatic vessels, in the zone A (B), in the zone B (E), in the zone C (H), close to a worm section (K) and for Podoplanin (C, F, I, L) in the zone A (C), in the zone B (F), in the zone C (I), close to a worm section (L) or for Lyve-1 (D, G, J, M) in the zone A (D), in the zone B (G), in the zone C (J), close to a worm section (M), to identify lymphatics vessels. Number of analysed nodules = 10. *O.v.*: *O. volvulus* section. Scales: A, bar = 300 µm; B,C,D,E,G,J,K,M bar = 25 µm; F,H,I,L, bar = 10 µm.

Blood and lymphatic vessels were identified on thin consecutive sections of *O. volvulus* nodules using monoclonal anti-CD31 antibody to detect endothelial cells (Figures 1A, 2B, 2E, 2H and 2K), and monoclonal anti- Lymphatic vessel endothelium receptor 1 (Lyve-1) (Figures 1B, 2D, 2G, 2J and 2M) or anti-Podoplanin (Figures 2C, 2F, 2I and 2L) antibodies to discriminate lymphatic endothelial cells. This revealed the presence of Lyve-1 and CD31 double positive lymphatic vessels as well as CD31 single positive blood vessels (Figure 1A–D).

Vascular distribution within the different zones of the nodules (Figure 2A) is summarised in Table 1. The vascularisation of the nodule was more developed in the dense infiltrate area (zone C). A comparison of the total number of vessels (CD31) with Lyve-1 and podoplanin positive vessels clearly demonstrated a dense network of lymphatics in addition to blood vessels. However, blood vessels were more numerous than lymphatic vessels in all three areas (Table 1). Podoplanin and Lyve-1 staining displayed similar results.

**Table 1:** Vessel density in zone A, B and C of the onchocerca nodule.

Vessels	zone A	zone B	zone C
CD31^+^ vessels	++	+++	++++
Podoplanin^+^ vessels	++	+	++
Lyve-1^+^ vessels	+	+	++

The densities of CD31^+^vessels and lymphatics in the nodule were determined in zone A, B and C. 0, no vessel section; +, 1 to 4 vessel sections; ++, 5 to 19 vessel sections; +++, 20 to 49 vessel sections; ++++, more than 50 vessel sections. Number of analysed nodules = 10.

Nodules were divided into three zones according to Edgeworth [16]. Zone A represents the outer fibrovascular capsule (Figure 2A). Zone B corresponds to the inner adult worm bundle with surrounding extracellular matrix interspersed with solitary cells (Figure 2A). Zone C is characterized by a dense cellular infiltrate surrounding and in contact with the adult worm (Figure 2A). These two last zones constitute the core of the nodule.

In detail, the zone A mainly contained middle-size, rounded CD31^+^ vessel sections; a few large- and small-size rounded vessel sections were also observed as well as few long flattened sections (Figure 2B). The zone B mostly contained middle- and large-size rounded vessel sections; few small-size rounded and long flattened vessel sections were also observed (Figure 2E). The zone C contained predominantly small-size rounded and short flattened vessel sections; few long flattened vessels were also seen in this dense cellular infiltrated area (Figure 2H). CD31^+^ vessels were often found close to the worm sections (Figure 2K). Blood vessels in the nodule were positive for collagen type IV, showing the presence of a basement membrane in these vessels (data not shown).

Regarding lymphatic vascularisation, the zone A mainly contained large flattened Podoplanin^+^ and Lyve-1^+^ lymphatic vessel sections and a few small-size rounded lymphatics (Figures 2C and 2D). The zone B was more diverse with irregular shapes of lymphatics ranging from middle-size rounded to long, flattened vessel sections (Figures 2F and 2G). The zone C mostly contained long to very long flattened sections (Figures 2I and 2J); few small-size rounded lymphatic vessel sections were also observed. Lymphatic vessels were observed often close to the worm sections (Figures 2L and 2M).

### Few Microfilariae Were Present within Lymphatic Vessels of the Nodule but Not of the Dermis

Besides being part of inflammatory processes, these newly formed lymphatics could be a pathway by which the microfilariae migrate out of the nodule into the dermis of patients. Thus after a specific staining of lymphatic vessels for Lyve-1 or podoplanin, we analysed the nodules and the dermis for the presence of microfilariae in the lumen of the lymphatic vessels.

**Figure 3:**
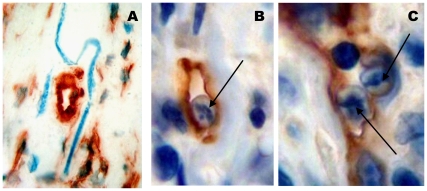
Presence of microfilariae in lymphatic vessels from the nodule. (A) Microfilariae (blue turquoise) in nodule close to a lymphatic section. (B & C) Sections of microfilariae (arrows) within lymphatic vessels in the nodules (Lyve-1 staining). Number of analysed nodules = 10. Scales: A, bar = 20 µm; B, C, bar = 10 µm.

Microfilariae were observed in all three zones but the majority was found in the core of the nodules. They were never identified in blood vessels. Some of them were present in close contact to the lymphatic vessels (Figure 3A). The presence of microfilariae within lymphatics was shown in the nodules presenting microfilariae (Figures 3B and 3C). The mean number of microfilariae per section of nodule was 41.5 ranging from 14 to 52. The percentage of microfilariae in the lymphatic vessels was 6.6% ranging from 3.8% to 11.4%.

**Figure 4:**
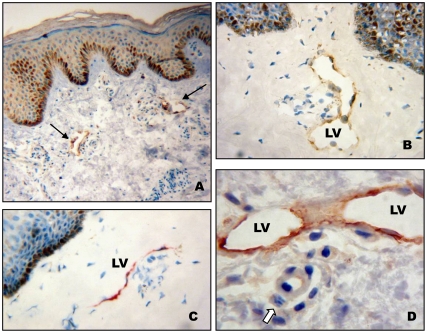
Localisation of microfilariae in the dermis of onchocercian patients. (A) Lyve-1 staining showing two dilated lymphatic vessels (black arrow)on a paraffin section (B) Lyve-1 staining revealing one dilated lymphatic vessel with mononuclear cells on a cryosection (C) Podoplanin staining showing a collapsed lymphatic vessel on a cryosection. (D) Microfilaria (white arrow) section in a perivascular space near to a lymphatic vessel. Number of analysed skin snips = 8. Scales: A,B,C,D, bar = 50 µm.

In the dermis, the mean number of microfilariae was 12.3±4.4 per section of skin snip (×100 magnification). Microfilariae were often close to the initial lymphatics but their presence inside them was not observed (Figure 4D). About 13% of microfilariae were observed in perivascular spaces, around lymphatic vessels, arterioles or nerve sections. Initial lymphatics were dilated (lymphatic ectasia) (Figure 4A) and some contained large mononuclear cells (Figure 4B); collapsed lymphatic vessels were also observed (Figure 4C).

### Onchocercomas Were Associated with a Molecular Profile of Lymphangiogenic and Angiogenic Markers

Angiogenic and lymphangiogenic cytokines and their receptors have been identified as key regulators of angiogenic and lymphangiogenic processes. Thus to determine the markers involved in vascularisation of onchocercoma, we analysed the gene expression of vascular endothelial growth factor (VEGF)-A, VEGF-C, VEGF-D, VEGF-R1, VEGF-R2, VEGF-R3, CXCL12, CXCR4, Angiopoietin-1 (Ang-1) and Angiopoietin-2 (Ang-2). The members of the VEGF family and their receptors, the Ang-1 and Ang-2, as well as the chemokine CXCL12 are central to the activation and maintenance of a neovascularisation.

**Figure 5:**
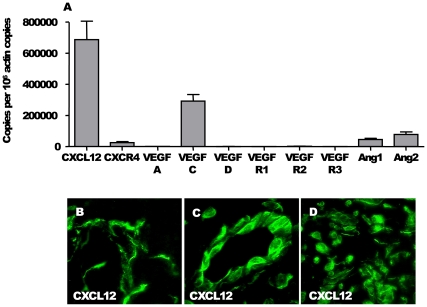
Angio- and lymphangiogenic markers in nodules. (A) A particular pattern of angio/lymphangiogenic factors was established by qPCR in nodules of onchocerciasis patients, characterized by the significant expression of CXCL12, CXCR4, VEGF-C, Angiopoietin-1 and Angiopoietin-2. Number of analysed nodules = 33. Data were analyzed using Prism version 4.0 (GraphPad) and presented as mean±SEM. (B-C-D) Expression of CXCL12 in nodule by immunofluorescence. Scales: B, bar = 20 µm.

Among them we found different levels of expression of VEGF-C, CXCL12, CXCR4, Ang-1, and Ang-2, with CXCL12 showing the strongest expression. Only a weak but significant mRNA expression of its unique receptor CXCR4 was measured. Elevated transcripts of VEGF-C, Ang-1, and Ang-2 were also detected but at a lower level than CXCL12 (Figure 5A). Other factors were not detectable.

The high expression of CXCL12 was translated into protein as we observed a strong staining for CXCL12 chemokine by cells in the nodule (Figure 5B–D). CXCL12 was detected on connective cells in the capsule (Figure 5B) and in the core of the nodule, on endothelial cells of blood and lymphatic vessels (Figure 5C) and on inflammatory cells (Figure 5D) in each of the analysed nodules.

### Infiltrated Macrophages Expressed the Lymphatic Endothelial Marker Lyve-1 and Were Associated with the Lymphatic Vasculature

**Figure 6:**
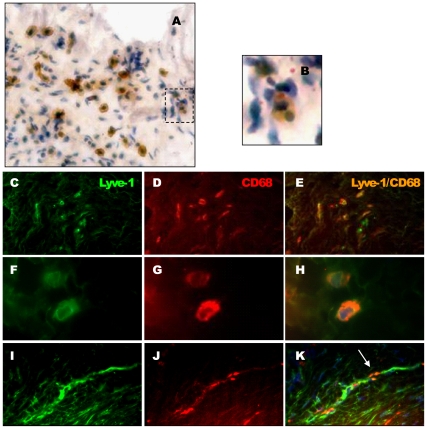
Lyve-1^+^ macrophages in nodule. (A) Individual Lyve-1^+^ mononuclear cells in the fibrous capsule of nodules. (B) Detail of a Lyve-1^+^ mononuclear cell phagocytising apoptotic neutrophil. (C–K) Double Immunostaining for Lyve-1/CD68 to establish the macrophage lineage of Lyve-1^+^ mononuclear cells: (C & F) Lyve-1^+^ mononuclear cells, (D & G) CD68^+^ macrophage, (E & H) merging C & D and F & G. (I–K) Incorporation of Lyve-1^+^ macrophages into lymphatics: (I) Lyve-1^+^ lymphatic vessel, (J) CD68^+^ macrophage, (K) merging I & J. Number of analysed nodules = 10. Scales: A,B,F,G,H, bar = 50 µm.

When staining onchocercomas for Lyve-1, we observed cells other than endothelial cells, being positive for Lyve-1. They were mononuclear and mainly localised in the capsular area (Figure 6A), barely in the zone B. Some of them showed a phagocytic phenotype (apoptotic neutrophils) suggesting that these cells could be macrophages (Figure 6B). Double staining for Lyve-1 and CD68, a known marker for macrophages in *Onchocerca* nodules, demonstrated the presence of macrophages expressing a lymphatic endothelial cell marker, Lyve-1, mainly in the capsule of the nodule (Figures 6C, 6D, 6E, 6F, 6G, and 6H). In addition double staining revealed the presence of Lyve-1+ macrophages in the endothelium lining of the lymphatic vessels suggesting their participation in neovascularisation (Figures 6I, 6J and 6K).

## Discussion

This study provides evidences for the presence of lymphatic vessels and pro-vascular factors within the human *Onchocerca* nodule as well as of microfilariae within lymphatic capillary beds. It further suggests that CD68+ macrophages expressing the lymphatic endothelial marker Lyve-1 are recruited to the site of lymphangiogenesis and these cells may participate to the vessel formation in the nodule, as it has been shown in various pathologies [54].

Only scattered information is known about the aetiology of onchocercoma; worms which are found in nodules are adults and the process of encapsulation of the female worms is unidentified. Authors in the early 1900 [25], [26] hypothesized that the *O. volvulus* female worm was trapped in a skin lymphatic vessel which would be not only the site of thrombosis but also the site of important remodelling with intense inflammatory response, as analysed since by several studies [12], [16], [27]. The female worm spends its lifespan in the nodule and releases microfilariae into an inflammatory environment. The general view is that nodules are highly vascularised with both blood and lymphatic vessels. Blood vascularisation was observed in onchocercomas [16], [18], [19]. However no data were available on a lymphatic network.

Imaging of lymphangiogenesis and angiogenesis requires robust and unambiguous markers of lymphatic and blood vessels. In recent years molecules specifically expressed on lymphatic and blood vessels were identified. We have used Lyve-1 [28] and Podoplanin [29] which are among the most useful markers for microscopic imaging of lymphatic vessels and CD31, a marker shared by lymphatic and blood vessels to analyse the presence of the two vascular systems in *Onchocerca* nodules (Figure 1). Both intensive blood and lymphatic vascularisation was established with different shape and size of vessel sections according to the zones of the nodule (Figure 2). In all cases, lymphatic vessels and worms were in close proximity and many collapsed lymphatics were found to surround the worm sections within the highly inflammatory infiltrated cell environment (Figure 2). A small proportion of microfilariae was observed within the lymphatic vessels of the nodule (6.6%) suggesting a possible role of the lymphatic system in the dissemination of *O. volvulus* microfilariae out of the nodule to the other parts of the body. However no microfilariae were found within the lymphatic vascular bed of the dermis, although we observed vascular changes in the dermis: some lymphatic vessels were dilated as evidenced by a well-distended wall. Previously, authors underlined the presence of such abnormal dilated capillary vessels and suggested a lymphatic phenotype [23], [30], [31], [32]. According to Bonucci *et al.* [23] some of these vessels contained microfilariae, a finding which could explain the dissemination of the parasite to other body areas. Observations on the human and cattle *Onchocerca* species [24], [33], [34], as well as on the experimental models of onchocerciasis *e.g. Monanema martini* in rodents have suggested that the microfilariae could migrate through the lymphatic system of the skin [35], [36], [37]. Indeed, dermic microfilariae of *Monanema martini* in rodents and those of *Cercopithifilaria johnstoni* in rats [38] were evidenced in the lymphatic vessels of the skin and in deeper localisations [36], [39]. In our hand, we regularly observed microfilariae in close contact with the lymphatic endothelial lining of the dermis, often in perivascular spaces (Figure 4D) but not inside the lymphatic vessel. Our findings do not exclude the possibility that microfilariae migrate through the lymphatic system but it is likely that it may not be their main pathway of migration.

The genesis of the blood and lymphatic vessels in the nodule may be the consequences of two separate or additive phenomena: first, neovascularisation could be induced by the worms themselves. Indeed, *O. volvulus* Activation associated Secreted Protein (Ov-asp) was found to induce angiogenic responses when injected into corneas of naive mice [40], [41]; second, neovascularisation could be induced by inflammation. There is increasing evidence for close association between inflammation, the microenvironment and tumour-associated neo-angiogenesis and lymphangiogenesis. Activated leukocytes in inflammatory sites produce a large number of growth factors including FGF, VEGF, and PDGF family members that might stimulate proliferation, migration and survival of endothelial cells [42].

The molecular profile of angiogenic and lymphangiogenic markers in nodules revealed a high expression of VEGF-C (Figure 5A) a potent inductor of both blood and lymphatic neovascularisation in tumor. VEGF-C is also found to be highly upregulated in patients infected with *Wuchereria bancrofti* [43]. Angiopoietins-1 and -2 were also put in light as angiogenic and/or lymphangiogenic markers in the nodules (Figure 5A). Indeed, the angiopoietins and their receptor Tie2 play an important role in vascular stabilization and pathologic neovascularization. Ang-1 activates Tie2 and functions as a positive regulator of remodeling and stabilization of blood vessels. Recently, Ang-1 and Ang-2 have been reported to regulate the formation of lymphatic vessels through Tie2 [44].

An even higher expression was found for the chemokine CXCL12 (Figure 5A) not only on mRNA level but also for the protein (Figure 5B). Its receptor CXCR4 could also be detected, however at a much lower mRNA (Figure 5A) and protein (data not shown) level. Many chemokines have a proangiogenic activity [45], in particular the ones signalling through CXCR1, CXCR2 and CXCR4 [46]. The CXCL12/CXCR4 couple is not only known for its proangiogeneic activities [47] but it is also a key element in endothelial cell recruitment to tumoral neovascularisation sites [48]. CXCL12 is hypothesised to exert its effect by attracting CXCR4-expressing circulation-derived endothelial precursor cells to sites where they subsequently contribute to neovascularisation. During this process, maturing cells lose precursor specific markers and express the same cell surface markers as mature endothelial cells involved in angiogenesis and likely in lymphangiogenesis.

In this study we demonstrated the presence of macrophages expressing the lymphatic endothelial marker Lyve-1 not only as isolated cells within the fibrous capsule but also incorporated into the new lymphatic vasculature within the core of the nodule (Figure 6). Lyve-1 positive macrophages have been shown to be essential for vessel formation in different pathological processes [49], [50], [51], [52], [53]. Macrophages apparently play a dual role in inflammation-induced lymphangiogenesis [54]: firstly they are able to secrete lymphangiogenic growth factors VEGF-C and VEGF-D that stimulate the growth of existing lymphatic endothelial cells [55], [56], and secondly they can trans-differentiate into lymphatic endothelial cells that incorporate to the lymphatic endothelium [57]. These macrophages expressing Lyve-1 also underline the fact that no perfect lymphatic marker has been found that works reliably in all species, tissues, vascular beds, and in all physiological and pathologic conditions. It is likely that the heterogeneity of expression of markers in lymphatic vessels reflects differences in the phenotype of endothelial cells. The recruitment of circulation-derived endothelial precursor cells [58] and/or the appositional growth of lymphatics are known mechanisms that contribute to lymphangiogenesis. However the role of macrophages should be considered, particularly in onchocercoma where they represent a large proportion of infiltrated inflammatory cells.

In conclusion, it is clear that the vascular system is critical in the modelling of the complex nodule microenvironment; the multifaceted axis CXCL12/CXCR4 and the growth factor VEGF-C appear to be essential to these angiogenic and lymphangiogenic processes. In lymphatic filariasis, doxycycline treatment decreases the production of VEGF-C [43]. The question arises whether doxycycline or other macrofilaricidal drugs affect lymphatic vessel formation. All together new therapeutic options targeting the neovascularisation processes deserve further investigation.

## Materials and Methods

### Patients, Onchocercomas and Skin Biopsies

Onchocercomas (n = 41) from patients in a hyper-endemic area in Ghana were randomly selected for this investigation. Selection criterion was presence of at least one fertile female within the nodule. Full details including ethical approval have been previously published [59]. The design of this randomized, placebo-controlled double-blind study was approved by the Committee on Human Research and Ethics of the School of Medical Sciences of the Kumasi University of Science and Technology (KNUST), Kumasi, Ghana as well as by the Ethics Committee of the University of Liver-pool, which acted as a control body since this work formed part of a European network funded by the European Commission. The study conformed to the principles of the Helsinki Declaration of 1974 (last amended 2004) and to Good Clinical Practice (GCP) standards. The trial is registered at Current Controlled Trials and has the registration number ISRCTN 71141922. All patients provided written informed consent for the collection of samples and subsequent analysis.

The skin snips were from 8 patients of Mbarembeng, Moungo, a region hyper-endemic for onchocerciasis, located in the Littoral province of Cameroon. The design of this study was approved by the National Ethics Committee, Yaoundé, Cameroon, as well as by the Ethics Committee of the French “Agence Nationale pour la Recherche”. The study conformed to the principles of the Helsinki Declaration of 1974 (last amended 2004) and to Good Clinical Practice (GCP) standards. The trial is registered at National Ethics Committee and has the registration number FWA IRB00001954. Two skin snips (one from each iliac crest) from every patient were made using a 2 mm Holth-type corneo-scleral punch. Parasitological examination for *O. volvulus* was done by microscopic examination for the presence of mf following incubation in 0.9% NaCl at room temperature.

### Immunohistochemical Staining for CD31, Lyve-1, and Podoplanin

Onchocercomas and skin snips were directly cryo-preserved in OCT compound at −80°C or were fixed in 10% buffered formaldehyde and embedded into paraffin.

Five-micrometer sections were obtained from OCT embedded tissues. Non-specific Ab binding was blocked by incubation in 20% normal mouse or goat serum (DAKO). Serial sections were stained for 60 minutes with mouse anti-human CD31 (DAKO) or goat anti-human Lyve-1 (4 µg/ml, R&D systems) or their respective IgG isotype control mAbs (R&D systems); donkey anti-mouse IgG (H+L) Alexa Fluor 594 (InVitrogen) and rabbit F(ab′)_2_ anti-goat IgG (H+L) FITC (Beckman Coulter) were used as secondary antibodies. Sections were counterstained with a DAPI Solution. Fluorescent microscope (Nikon) was used with the Envision software.

CD31 and Podoplanin (0.2 µg/ml, AbD Serotec) were also visualized with the EnVision+ Dual link system-HRP followed by AEC+ substrate-chromogen system (DAKO). Lyve-1 was visualized with the Vectastain kit followed by DAB substrate-chromogen (Vector). Sections were counterstained with hematoxylin and mounted for microscopy.

Lyve-1 staining was also performed on five-micrometer sections from paraffin embedded tissues. Tissues were cut, deparaffinized, and rehydrated through graded alcohols. Antigen retrieval was performed by heating the slides for 20 minutes in a pH 6 citrate buffer at 98°C. Endogenous peroxidase was quenched for 10 minutes with peroxidase blocking reagent (DAKO). The above protocol was used for the antibody staining and its visualization.

Hematoxylin-eosin staining was also performed to reveal general organisation of onchocercomas.

### Vascular Organisation of the Nodule

The nodules were divided in 3 areas according to Edgeworth [16]. Zone A represented the outer fibrovascular capsule. An inner area is composed of spaced infiltrated cells (Zone B) and dense cellular infiltrate (zone C) surrounding the worm (Figure 1A). Immunostaining density of the CD31^+^, Podoplanin^+^ or Lyve-1^+^ vessels (0, no vessel section; +, 1 to 4 vessel sections; ++, 5 to 19 vessel sections; +++, 20 to 49 vessel sections; ++++, more than 50 vessel sections) was evaluated by light microscopy (x20 objective). Vessel sections were described as rounded (small, middle or large-size) when lumen was present, or flattened (short or long) when lumen was almost absent to totally absent.

### Identification of Microfilariae within the Lymphatic Vessels

Microfilariae were visualized by hematoxylin counterstaining and their presence within lymphatic vessels was analysed on Lyve-1^+^ sections from onchocercomas and skin snips. Microfilariae were numerated on whole Lyve-1^+^ sections from nodule with high magnification (x50). Microfilariae sections close together were considered as one microfilaria. Percentage of microfilariae within lymphatics was reported.

### Double Immunofluorescence for Lyve-1/CD68

Non-specific Ab binding was blocked by incubation in 20% normal donkey serum (DAKO, France). A Lyve-1/CD68 immunofluorescence double stain of nodule cryo-sections was performed. An antibody directed against Lyve-1 (4 µg/ml, R&D systems) was applied for 60 minutes to the sections. FITC Donkey anti-Goat IgG (1.5 µg/ml, Jackson IR) was used to visualize binding of the first antibody. Non-specific Ab binding was blocked by incubation in 20% normal goat serum (DAKO, France). The sections were then incubated for 60 minutes with a monoclonal antibody against CD68 (DAKO). TRITC Goat anti-mouse IgG (1∶100, Nordic Immunology) was used to visualize binding of this second antibody. Hoechst (4 µg/ml, Sigma) was used to stain the nuclei. Fluorescent microscope (Nikon) was used with ACT-1C software.

### Immunofluorescence Staining for CXCL12 and CXCR4

Non-specific Ab binding was blocked by incubation in 20% normal goat serum (DAKO, France). The sections were stained for 60 minutes with mouse anti-human CXCL12 mAb (clone 79018) or mouse anti-human CXCR4 (clone 12G5) or their mouse IgG isotype control mAbs obtained from R&D systems; mAb F(ab′)_2_ fragment goat anti mouse IgG (H+L) FITC was used as a secondary antibody. Fluorescent microscope (Nikon) was used with Envision system.

### Nodule RNA Extraction, cDNA Synthesis and Real-Time qPCR for Vascular Markers

Total RNA was extracted from 33 nodules. The cDNA was generated by reverse transcription using Omniscript-RT kit (Qiagen) and random primers in a 20 µl reaction containing 1 µg of total RNA, which was pretreated with RNase-free DNase I (Qiagen, CA) to eliminate contaminating genomic DNA.

Real-time PCR was performed on Rotorgene 6000 (Corbett Research). qPCR was performed in 10 µl consisting of 1 µl PCR buffer, 0.2 µM dNTPs, 0.1 µl Sybr Green I (1∶1000 in DMSO, Roche), 0.25 U Hotstar Taq polymerase (Qiagen) and 2 µl cDNA template. Primers used in qPCR (VEGF-A, VEGF-C, VEGF-D, VEGF-R1, VEGF-R2, VEGF-R3, CXCL12, CXCR-4, Angiopoietin 1, Angiopoietin 2) are listed in Table S1. Gene expression levels were calculated relative to the house-keeping gene β-actin. Data were analyzed using Prism version 4.0 (GraphPad) and presented as mean ± SEM.

## Supporting Information

Table S1 (0.04 MB DOC)
