# Supplementary material for: Lymphatic Vascularisation and Involvement of Lyve-1+ Macrophages in the Human Onchocerca Nodule
Source: PLoS One. 2009 Dec 9;4(12):e8234. doi: 10.1371/journal.pone.0008234 (PMC2784295; doi:10.1371/journal.pone.0008234)
Supplement: Table S1 — (0.04 MB DOC) [file pone.0008234.s001.doc]

**Supporting Document 1. Primer sequences.**

| Target gene | Forward 5' to 3' | Reverse 5' to 3' |
| --- | --- | --- |
| CXCR4 | acc aca gtc atc ctc atc ctg | cac acc ctt gct tga tga ttt |
| CXCL12 | aga gct cgc ttt gag tga ctg | atg tca cct tgc caa cag ttc |
| VEGF | gct tgg gga gat tgc tct act | ctc tga ccc cgt ctc tct ctt |
| VEGF-C | gtc ttt cct gaa cca tgt gga | atc ttg gct gtt tgg tca ttg |
| VEGF-D | cgt tgc gag tgt gtc tgt aaa | ttg cac atg gtc tgg tat gaa |
| VEGFR-1 | tga cct gga gtt acc ctg atg | gat ggt cca ctc ctt aca cga |
| VEGFR-2 | tga ccc caa att cca tta tga | act gtc cgt ctg gtt gtc atc |
| VEGFR-3 | tcg gag gag ttt gag cag ata | ttg tcc tcc tgg ttc ctc ttt |
| Angiopoietin 1 | agt ggc tgc aaa aac ttg aga | gtc tga gag agg agg ctg gtt |
| Angiopoietin 2 | gtg act gcc acg gtg aat aat | ttg tgg tgt gtc ctg att tga |
| Actine b | gat gag att ggc atg gct tta | aat gtg caa tca aag tcc tcg |

**Footnotes**

**Author contributions.** CM, SS, AH, OB conceived and designed the experiments. TA, GD, CM performed the experiments. AYD, YMD, MB, SW, VM, SM, OA contributed reagents/materials/analysis tools. CM, SS, AH, OB analyzed the data. OB and CM wrote the paper.

**Financial support:** This work was supported by European Community grant INCO-CT-2006-032321. T. Attout was supported by a postdoctoral fellowship from Région Ile de France.

The funders had no role in study design, data collection and analysis, decision to publish, or preparation of the manuscript.

**Potential conflicts of interest:** none reported.
